# RVD: a command-line program for ultrasensitive rare single nucleotide variant detection using targeted next-generation DNA resequencing

**DOI:** 10.1186/1756-0500-6-206

**Published:** 2013-05-23

**Authors:** Anna Cushing, Patrick Flaherty, Erik Hopmans, John M Bell, Hanlee P Ji

**Affiliations:** 1 Division of Oncology, Department of Medicine, Stanford University School of Medicine, Stanford, CA 94305, USA; 2 2Department of Biochemistry, Stanford University School of Medicine, Stanford, CA 94305, USA; 3 Stanford Genome Technology Center, Stanford University, Palo Alto, CA 94304, USA; 4 Department of Biomedical Engineering, Worcester Polytechnic Institute, Worcester, MA 01605, USA

**Keywords:** Next-generation sequencing, Rare variant detection, Genotyping

## Abstract

**Background:**

Rare single nucleotide variants play an important role in genetic diversity and heterogeneity of specific human disease. For example, an individual clinical sample can harbor rare mutations at minor frequencies. Genetic diversity within an individual clinical sample is oftentimes reflected in rare mutations. Therefore, detecting rare variants prior to treatment may prove to be a useful predictor for therapeutic response. Current rare variant detection algorithms using next generation DNA sequencing are limited by inherent sequencing error rate and platform availability.

**Findings:**

Here we describe an optimized implementation of a rare variant detection algorithm called RVD for use in targeted gene resequencing. RVD is available both as a command-line program and for use in MATLAB and estimates context-specific error using a beta-binomial model to call variants with minor allele frequency (MAF) as low as 0.1%. We show that RVD accepts standard BAM formatted sequence files. We tested RVD analysis on multiple Illumina sequencing platforms, among the most widely used DNA sequencing platforms.

**Conclusions:**

RVD meets a growing need for highly sensitive and specific tools for variant detection. To demonstrate the usefulness of RVD, we carried out a thorough analysis of the software’s performance on synthetic and clinical virus samples sequenced on both an Illumina GAIIx and a MiSeq. We expect RVD can improve understanding the genetics and treatment of common viral diseases including influenza. RVD is available at the following URL:http://dna-discovery.stanford.edu/software/rvd/.

## Background

Next generation sequencing (NGS) is currently used in a research setting to discover novel mutations in cancer, viral, and environmental samples. As the cost of sequencing decreases, this technology is increasingly used to assess genetic diversity both for basic research as well as translational applications in human diseases. Citing an example of a clinical application, rare variants occurring in pathogens such as viruses or cancer may lead to therapeutic resistance. However, to ensure that causative mutations do not evade detection in such a clinical setting, we must improve the resolution, sensitivity and specificity of available algorithms to detect such mutations. We present a computational tool to detect very rare mutations in targeted clinical samples, available for use with multiple next-generation sequencing technologies.

The detection level of current algorithms is limited by the inherent error rate in next generation sequencing technologies, generally quoted as 1-3% [1,2] or 0.25% in [3]. CRISP [4] reports to detect variants in large pooled data sets at 2% MAF on an Illumina GA platform but with only 86.3% sensitivity and 97% specificity. SPLINTER [5], a recent improvement on SNPSeeker, reliably detects mutations at 0.1% MAF using large deviation theory but requires both a positive and negative control. SNVer [6] outputs significance p-values for the likelihood of a variant at each position in a sample but does not take into account site-specific error. Mild et al. [7] reproducibly detects variants in HIV with MAF as low as 0.27% but is limited by its application on a 454 FLX Platform. By significantly changing the sample preparation technique, Schmitt et al. report a resolution of 1x10^-9^[8].

**Figure 1:**
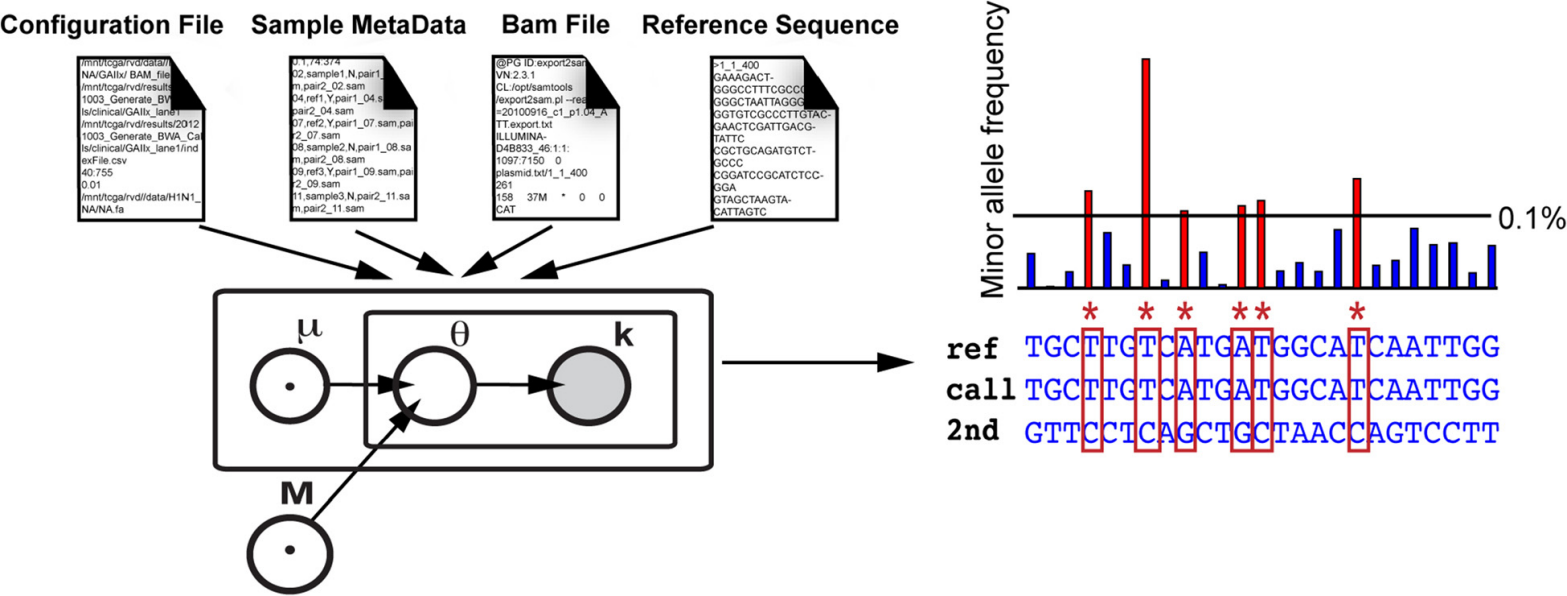
**Workflow of Rare Variant Detection (RVD) Application.** The standalone application requires four inputs: a configuration file containing directory locations, base quality and resolution thresholds, and region of interest information, a metadata information file with sample-specific information, a reference sequence file, and BAM format files with short read sequencing data for each sample. A beta-binomial prior distribution is applied to estimate the error rate of reference replicates that are sequenced alongside samples. In the figure, θ is the binomial parameter that represents the true error rate at a single position in a single experimental replicate. The variable θ has a beta distribution with parameters {M_0, μ} where μ is the prior error rate estimated at the given position and M_0 is the experimental precision of the error rate. The output of the application is a tab-delimited text file containing, for each position in the reference sequence, an estimate of the minor allele frequency (MAF) in the sample and a statistical call for significance. RVD is able to detect single nucleotide variants with minor allele frequency as low as 0.1%.

Recently, we demonstrated an algorithm for detecting very rare mutations in clinical samples from targeted next-generation sequencing data [3]. The implementation of the original method required access to MATLAB as well as extensive preprocessing to convert the data to a usable format. Also, we used filtering that allowed no more than two mismatches between each read and the reference sequence, thus limiting the usefulness of the algorithm for longer-read (>50 base pair) data sets. Here, we provide an implementation that operates directly on BAM formatted data files and is available as a command line program. This program outputs a simple table of called variants in one computational step (Figure 1). We also increase the usefulness of our method for sequence data of longer read length by implementing filtering of sequence data on base quality score rather than on number of sequence mismatches. We demonstrate our algorithm on samples sequences in multiple lanes of the Illumina GAIIx platform and on samples sequenced on the Illumina MiSeq platform.

### Implementation

RVD is available as a command-line program for the Unix platform and requires only access to samtools and the MATLAB compiler runtime (MCR), a free utility provided with the application package. RVD users prepare a configuration information file containing the region of interest, resolution and base quality thresholds, and reference sequence information as well as a sample meta-data file containing sample information. The RVD program package is available at http://dna-discovery.stanford.edu/software/rvd/ and a detailed user guide is provided in Additional file 1. RVD implements the following basic steps.

### Generate depth tables from user input BAM files

RVD sorts BAM files and converts them to samtools pileup format files. The pileup files are then used to generate depth tables containing base-specific coverage at each position in the sequence. Phred scores are calculated for each base from the pileup file base quality scores using the ASCII offset appropriate for the data set (33 as default or may be set manually as an optional parameter by user). Unmapped reads are removed and the remaining reads are filtered to remove alignments below a user-defined quality threshold.

### Estimate site-specific reference error distribution

RVD estimates the context-specific error rate based on the number of non-reference reads in each of the sequenced reference samples. A beta-binomial model is used to calculate a reference error rate distribution for each position in the sequence. One of the parameters of this model, M_0_, is used to estimate the experimental precision of sample preparation. Experimental precision, and thus performance of the algorithm, can be maximized if the same preparation techniques, batch of reagents, and sample sequencing flow cell are used to prepare and sequence the reference and the samples (see Sample Preparation Requirements).

### Test samples on reference error rate

RVD compares site-specific sample error rates to the estimated reference error distributions using a p-value of 1x10^-6^ to call variants.

### Call variants to output

RVD filters calls based on the resolution threshold and outputs a simple call table for the region of interest of each sample. RVD also outputs a single text file containing information about the sequencing process error calculated during analysis.

## Methods for sample preparation

To test the effectiveness of our rare variant detection method in clinical applications, we applied it to both the synthetic and clinical data sets reported in [3]. The synthetic DNA samples consisted of ~400 base-pair long reference and sample sequences that were synthesized in-vitro. The sample construct contained 14 single nucleotide changes at known positions compared to the reference. The sample and reference DNA were combined at known molar fractions: 0%, 0.1%, 0.3%, 1%, 10%, and 100% and sequenced in triplicate on the Illumina GAIIx to determine the accuracy of the method.

Twelve clinical samples were obtained from nasopharynegeal swabs of patients infected with H1N1 influenza and sequenced alongside three H1N1 neuraminidase reference replicates. To test the applicability of our method to novel technologies, we sequenced the same clinical libraries on a MiSeq platform. We sequenced one clinical sample (BN1) in replicate in multiplex with each platform and between platforms, allowing us to compare intra- and inter-platform reproducibility.

### Sample preparation requirements

The algorithm is designed to account for sequencing variation by repeated observation of the reference sequence. Consequently, it is important to control the protocols of sample storage and preparation for both the samples of interest and the reference samples. In particular, we recommend identical extraction and storage of nucleic acids [9], starting amounts of nucleic acids, library preparation and PCR protocols [10]. To achieve the optimal detection threshold for variants, we also find that the sample and reference should be sequenced on the same flow cell, though this requirement is not mandatory (see Table 1).

**Table 1:** Detecting rare synthetic variants across multiple lanes

**Mutant fraction of sample**	**0.1%**	**0.3%**	**1%**	**10%**	**100%**
Sequence lane	1	2	2	3	3
Sensitivity	100%	100%	100%	100%	100%
Specificity	98.8%	100.0%	99.9%	99.8%	100%

When the reference and sample are sequenced in different lanes, rare (>0.3%) variants are still called with high sensitivity and specificity. A 0.01% resolution threshold and 30 base quality threshold were used, and the reference was sequenced in Lane 1.

## Results and discussion

### Setting the resolution threshold

**Figure 2:**
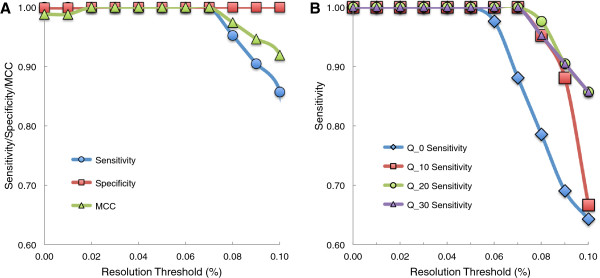
**Measuring sensitivity, specificity, and Matthews correlation coefficient (MCC) for optimal choice of resolution threshold and base quality threshold.** Analysis was done averaging sensitivity, specificity, and MCC across three 0.1% synthetic mixture DNA replicates sequenced on a GAIIx platform. **(A)** With a base quality threshold of 30, the specificity increases slightly from 0.999 to 1 as the resolution threshold is increased from 0.01% to 0.1%. More drastically, the sensitivity decreases at a threshold greater than 0.05% from near 1 to 0.857 at 0.1%. The MCC statistic that combines these two measures and adjusts for unequal distribution of true positives and true negatives peaks a range of 0.02% - 0.07%, indicating an optimal resolution threshold. **(B)** As the base quality threshold is increased from 0 to 30, RVD sensitivity becomes less dependent on resolution threshold. Thus, a higher base quality threshold may improve resolution of the algorithm.

By testing a range of resolution thresholds, we find that an optimal threshold to jointly maximize sensitivity and specificity is ½ of the desired MAF detection level. Flaherty et al. reported 98% specificity and 100% sensitivity on the 0.1% synthetic mixture in the previous version of this algorithm [3]. We tested dependence of specificity and sensitivity on resolution threshold by computing an average specificity and sensitivity across the three synthetic DNA replicates using a base quality threshold of 30. We find the sensitivity decreases from 100% to 85.7% and specificity increases from 99.9% to 100% as the resolution threshold is increased from 0.01% to 0.1% (Figure 2A).

We repeated the resolution threshold scan for the 0.3%, 1%, 10%, and 100% synthetic mixtures and find that the optimal resolution threshold for all mixtures is consistently half of the MAF (Additional file 2: Figure S1). For example, to optimally detect a MAF of 1%, a resolution threshold of 0.5% should be used. If the objective is only to maximize specificity, a higher threshold should be used. If the objective is only to maximize sensitivity, a lower threshold should be used.

### Setting the base quality threshold

The base quality threshold and resolution threshold can be adjusted to optimize the performance of the algorithm (Figure 2B). Increasing the base quality threshold had no effect on sensitivity at low (<0.05%) resolution thresholds. However, at a higher (0.1%) resolution threshold, increasing the base quality threshold from 0 to 30 drastically increases the sensitivity of the algorithm from 64% to 86%. Thus, as base quality threshold increases, RVD sensitivity becomes less dependent on the resolution threshold.

Changing the base quality threshold does not significantly change the specificity of the algorithm. A base quality threshold of 30 provides the best performance with specificity of 99.9% at a 0.01% resolution threshold and 100% specificity for all resolution thresholds greater than 0.01%. The lowest specificity, 99.0%, occurs at a base quality threshold of 10 and no resolution threshold. Because this decrease in specificity between base quality thresholds of 10 and 30 is not significant, the choice of base quality threshold will likely not affect the overall specificity of RVD.

As the base quality threshold is increased from 0 to 30, the average reference error rate in the synthetic samples is reduced from 0.26% to 0.02%. Further, the maximum base quality in the synthetic data set is 38 compared to 41 in the clinical GAIIx and MiSeq sequence samples. This variation in base quality between runs suggests the optimal base quality threshold may be run-dependent.

### Sequencing reference and sample in different lanes

Sequencing the reference and sample in different lanes has little effect on the resolution of the method. We sequenced the reference in lane 1 in multiplex with the 0.1% synthetic mixture while the 0.3% and 1% mixtures were sequenced in lane 2 and the 10% and 100% mixtures were sequenced in lane 3. We find that for variants with 0.3% MAF and above, sequencing the reference in a different lane still allows for high sensitivity and specificity (Table 1). Thus, users can maximize capacity by sequencing the reference replicates in only one lane while sequencing multiple lanes of clinical samples, with a moderate decrease in resolution.

### Comparison of calls on MiSeq and GAIIx platform

When identical clinical libraries were sequenced on the Illumina MiSeq and GAIIx platforms, RVD was able to detect many rare (<1%) mutations that were called on the GAIIx (Additional file 2: Figures S2 S3). There was an increase in the site-specific standard deviation (0.005% vs 0.09%) but a slightly lower average error rate (0.055% vs 0.05%) in the MiSeq data at a base quality threshold of 30. The MiSeq run generated 9.5x10^6^ 37 bp aligned single-end reads for an average coverage of 11,723 across the data set with average sample-specific coverage ranging from 1,854 to 34,976 reads. The GAIIx, comparatively, produced an average coverage of 103,130 with sample-specific coverage ranging from 13,535 to 341,523 reads.

**Figure 3:**
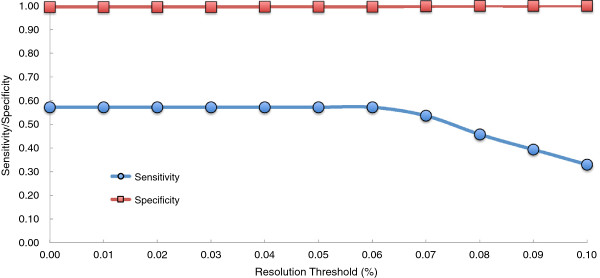
**Sensitivity and specificity to detect rare variants on the MiSeq platform.** Four replicates of sample BN1 were run on the GAIIx (two each in lanes 1 and 2) and variant calls made on all four replicates at a resolution threshold of 0.01% (10x lower than a previously reported lower threshold) and a base quality threshold of 30 were considered “true” variants. Two MiSeq BN1 replicate calls were each compared to the set of true variants, and the sensitivity and specificity was averaged across the MiSeq replicates. MiSeq shows 43% loss of sensitivity compared with GAIIx but no significant loss of specificity. When the resolution threshold for the MiSeq calls was increased from 0.01% to 0.1%, the sensitivity decreased as expected.

We identified a set of concordant variants as those variants that were called similarly on all four GAIIx BN1 replicates (two each in lane 1 and 2). This set was used to estimate sensitivity and specificity of MiSeq calls. The sensitivity is 57.1% and the specificity is 99.5% with a quality score threshold of 30 and no resolution threshold (Figure 3).

There was a 58.2% concordance of BN1 variant calls within the MiSeq platform compared with 79.1% concordance within the GAIIx with no minimum resolution threshold and a quality score threshold of 30. We find a 42.5% concordance between BN1 variants called with consensus on the GAIIx and those called with consensus on the MiSeq. Lower levels of concordance and sensitivity may be due to the fact that this data was collected on an early iteration of the MiSeq platform with shorter reads and lower average qualities than newer MiSeq data. In addition, this early Miseq run had 10-fold lower depth of coverage compared to the GAIIx.

## Conclusions

We provide here a tool for identifying rare mutations directly from targeted resequencing data sets. The improved resolution to detect rare mutations using this tool can aid in our understanding of the relationship between rare, novel mutations that occur in samples demonstrating genetic heterogeneity. For example, this genetic diversity is seen in infectious viruses such as HIV and HCV. In the future, we plan to investigate RVD’s statistical power using lower depth (<10,000) samples for use on longer genomic regions. These next steps will allow us to apply RVD’s high sensitivity and specificity to improve understanding of rare mutations in cancer genes and the complex genetics involved in cancer tumor evolution.

## Availability and requirements

Project name: RVD

Project home page: http://dna-discovery.stanford.edu/software/rvd/

Operating system(s): Linux, Mac OS X

Programming Language(s): MATLAB

Other requirements: Samtools 0.1.18, MATLAB compiler runtime version 7.17 (provided in the program package), x11

License:

Any restrictions to use by non-academics: None

## Abbreviations

MAF: Minor allelic fraction; BAM: Binary alignment map; HIV: Human immunodeficiency virus; HCV: Hepatitis C virus

## Competing interests

The authors declare they have no competing interests.

## Authors’ contributions

PF and AC drafted the manuscript and performed statistical analysis of the program. PF designed and wrote the software algorithm. AC participated in writing the software and wrote the user guide. EH carried out the sequencing and sample preparation. JMB participated in the sequence alignment. HJ came up with the project and participated in its design, coordinated and oversaw the project, and helped draft the manuscript. All authors read and approved the final manuscript.

## Supplementary Material

Additional file 1 Containing a detailed user guide explaining how to download and run the RVD program. Click here for file

Additional file 2 Containing supplementary figures. Click here for file
