# Supplementary material for: RVD: a command-line program for ultrasensitive rare single nucleotide variant detection using targeted next-generation DNA resequencing
Source: BMC Res Notes. 2013 May 23;6:206. doi: 10.1186/1756-0500-6-206 (PMC3695852; doi:10.1186/1756-0500-6-206)
Supplement: Additional file 1 — Containing a detailed user guide explaining how to download and run the RVD program. [file 1756-0500-6-206-S1.pdf]

ALGORITHM USER GUIDE FOR RVD

The RVD program takes BAM files of deep sequencing reads in as input. Using a Beta-Binomial model, the algorithm estimates the error rate at each base position in the reference sequence. For each sample, the relative difference between the reference and sample error rate is calculated and tested against the null distribution estimated from the model. The reproducibility of this algorithm has been tested when samples and reference are sequenced in the same lane as well as in different lanes. Data from these reproducibility tests show the algorithm is able to reliably call variants as low as a minor allele fraction of 0.1%. Deployed RVD runs in approximately 11 minutes per clinical sample (ex. 131 minutes for twelve 700-basepair clinical samples and 3 references) on an AMD Opteron 8382 processor.

REQUIREMENTS FOR RVD:

Dependencies:

❑ Access to MATLAB **OR** MATLAB Compiler Runtime v717 (free compiler provided in program package)
❑ X11 (for MATLAB Compiler Runtime installation, comes standard with most MacOSX systens)
❑ Samtools (tested with version 0.1.18)

Data files:

❑ BAM format sequence data files
- Single end aligned reads of continuous region of interest
- High depth coverage
- Three reference replicates sequenced alongside samples

❑ FASTA format reference sequence file (should be the same reference used to align samples)

User-created files:

❑ Configuration file
❑ Sample index file

DIRECTIONS FOR INSTALLING AND RUNNING RVD:

If you do not have samtools 0.1.18, download and install the program package:

❑ Download samtools 0.1.18 from http://sourceforge.net/projects/samtools/files/
❑ untar the program package:

    tar –zxvf samtools-0.1.18.tar.bz2

❑ enter the samtools directory

    cd samtools-0.1.18

❑ make the samtools program

    make clean
    make all

❑ copy all of the samtools binaries to a destination directory in your PATH (/usr/local/bin, /opt/bin, etc)

    cp bin/* destination_location

If you have MATLAB and would like to run the program directly in MATLAB:

❑ Download the program files

The algorithm is run using the main program file, RVD.m, in MATLAB with the following command:
RVD [CONFIGURATION FILE]

If you do not have MATLAB or would like to run the program from the terminal:

❑ Executable binaries are available for mac and 64-bit Linux. Download the zipped package file appropriate for your operating system:

❑ Unzip the package into a directory of your choice.

Once the package is unzipped, the RVD program is now installed!

In order for the command-line program to work correctly **it is essential to reference MATLAB compiler runtime version 7.17 in running the program.** If MATLAB R2012a is installed on your machine, referencing the location of its installation is equivalent to referencing MCR v7.17 and thus the MCR does not need to be installed. Referencing a different version of the MCR or a version of MATLAB other than R2012a will cause an error message.

❑ Install the MCR 7.17 package:

A zipped installer called `MCRInstaller.zip` will be in the directory. This contains the standalone MATLAB libraries needed by the executable.

❑ Unzip the MCRInstaller.zip into a folder called `MCR`.

❑ Run `install` in the MCR folder to install those libraries. Keep track of where that library is installed because you will need to specify the root location when running the RVD executable. (From a MAC, you may choose to click manually on the InstallForMaxOSx application icon to start the install)

❑ Some systems may require you to set the LD_LIBRARY_PATH (or DYLD_LIBRARY_PATH for maxi64) and XAPPLRESDIR environmental variables. The best way to check this is to run the program. Even if the environmental variables are not manually set, when the program is run it should immediately print out "Seting up environment variables" followed by the LD_LIBRARY_PATH (or DYLD LIBRARY_PATH) location as shown below:

LD_LIBRARY_PATH is
.:<mcr_root>/v717/runtime/glnxa64:<mcr_root>/v717/bin/glnxa64:<mcr_root>/v717/sys/os/glnxa64:<mcr_root>/v717/sys/java/jre/glnxa64/jre/lib/amd64/native_threads:<mcr_root>/v717/sys/java/jre/glnxa64/jre/lib/amd64/server:<mcr_root>/v717/sys/java/jre/glnxa64/jre/lib/amd64

DYLD_LIBRARY_PATH is
.:<mcr_root>/v717/runtime/maci64:<mcr_root>/v717/sys/os/maci64:<mcr_root>/v717/bin/maci64:/System/Library/Frameworks/JavaVM.framework/JavaVM:/System/Library/Frameworks/JavaVM.framework/Libraries

The <mcr_root> is the directory where MCR is installed on your computer. If the variable output does not reflect the above, the program will give an error message about installed libraries and the program will quit immediately. If this happens you will need to set the environmental variables using the following commands with <mcr_root> again being the directory where you have installed MCR:

For LINUX:
setenv LD_LIBRARY_PATH

$LD_LIBRARY_PATH:<mcr_root>/v717/runtime/glnxa64:<mcr_root>/v717/bin/glnxa64:<mcr_root>/v717/sys/os/glnxa64:<mcr_root>/v717/sys/java/jre/glnxa64/jre/lib/amd64/native_threads:<mcr_root>/v717/sys/java/jre/glnxa64/jre/lib/amd64/server:<mcr_root>/v717/sys/java/jre/glnxa64/jre/lib/amd64

setenv XAPPLRESDIR <mcr_root>/v717/X11/app-defaults

For MAC:
setenv DYLD_LIBRARY_PATH
$DYLD_LIBRARY_PATH:<mcr_root>/v717/runtime/maci64:<mcr_root>/v717/sys/os/maci64:<mcr_root>/v717/bin/maci64:/System/Library/Frameworks/JavaVM.framework/JavaVM:/System/Library/Frameworks/JavaVM.framework/Libraries

setenv XAPPLRESDIR <mcr_root>/v717/X11/app-defaults

Alternatively instead of setenv you may use the command:
VARIABLE = $VARIABLE\:new_path
export VARIABLE
You may check the value stored in each variable using:
echo $VARIABLE

The software is now installed and the executable binary for the algorithm is called from `run_RVD_system.sh (run_RVD_glnxa64.sh for Linux and run_RVD_mac.sh for mac).` The syntax for running the algorithm is:
$ sh run_RVD_system.sh [<mcr_root>/v717] [CONFIGURATION FILE]

example: sh run_RVD_glnxa64.sh /usr/local/MATLAB_Compiler_Runtime/v717 RVD_ex/configFile.txt

CREATING REQUIRED FILES FOR RVD:

☐ **Create a configuration file. The format for the configuration file is as follows:**

The configuration file has 6 lines that define file locations and meta information for the algorithm. The following should each be on their own lines:

1. **data path**: the file path to the directory where the BAM files are stored.

2. **results path**: the file path to the directory where you want the results charts to be stored

3. **index file name**: the path to the index file you must create in order to provide the program with adequate data about the samples to be analyzed. See below for more information about the formatting of the index file.

4. **ROI**: the region of interest with respect to the BAM file. 30:600, for example, would cause bases 30-600 of the sample to be analyzed by the algorithm. The algorithm also has its own region of interest filtering

such that the edges of the region of interest will be trimmed to ensure that depth is at least 1000 at the edges of the region to be analyzed.

5. **resolution threshold:** the resolution threshold is a cutoff value that is applied to the minimum centered error percent, a measure of the relative difference between the expected error rate at each position and the error rate actually seen in the sample. Only positions that have been called by the algorithm to be variants and whose centered error percent is above the resolution threshold are included as variants in the output call tables.

Based on empirical analysis of synthetic samples we find an optimal resolution threshold for all mixtures is approximately half of the desired minor allele frequency (MAF) detection level. For example, a 0.05 resolution threshold should be used to detect 0.1% MAF mutants. If the objective is only to maximize sensitivity, a lower threshold should be used. If the objective is only to maximize specificity, a higher threshold should be used.

6. **reference sequence file**: the file path to the reference sequence fasta file that was used to align the BAM sequence data files.

7. **base quality threshold:** the base quality threshold is used to filter low quality alignments before performing variant detection. A base quality threshold of 30 is suggested for high sensitivity and specificity. Higher base quality thresholds decrease the reference error estimated by the algorithm and thus increase sensitivity to vey rare variants at higher resolution thresholds but do not significantly increase specificity.

8. **ASCII base quality offset (optional):** an optional parameter to set the ASCII offset used to determine the phred base quality scores from the pileup files. If this line is present, the value in the file will be used as the ASCII offset. If no value is present, the default value is set to 33. Most recently generated sequencing data (Illumina 1.8 and later) uses an ASCII offset of 33 (Sanger format). Most older sequencing data (Illumina earlier than 1.8) uses Solexa format quality scoring and thus requires an ASCII offset of 64.

An example configuration file is shown below:

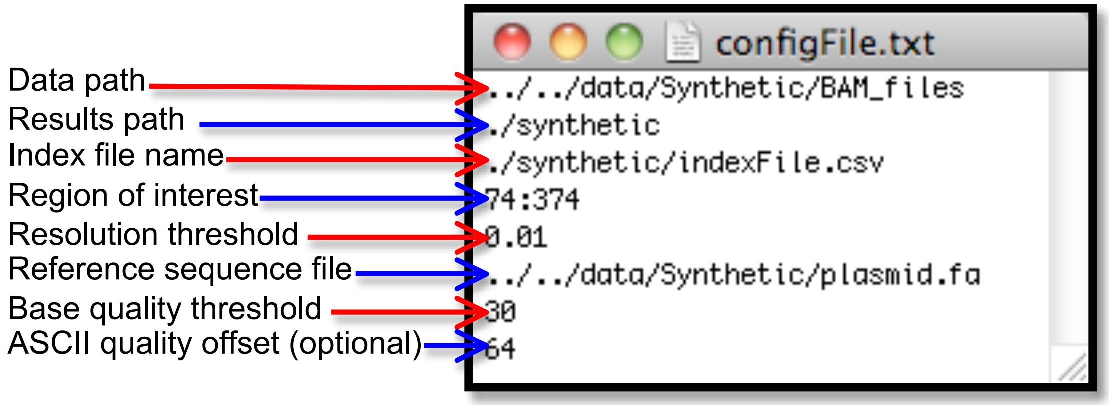

☐ **Create a sample index file. The format for the index file is as follows:**

The program also requires comma-separated file that defines the meta-information for each BAM file where each line is in the following format:
tag, sampleName, isReference, pair1BamFile, pair2BamFile

tag is the tag used to sequence the given sample. Tag can be a number, 3 base sequence, or any other variable the user chooses to distinguish.

sampleName allows the user to name the sample.

isReference determines whether the sample is read as a reference sample (Y) or a non-reference sample (N).

pair1BamFile is the name of the bam file containing information for the pair1 read.

pair2BamFile is the name of the bam file containing information for the pair2 read.

An example index file is show below:

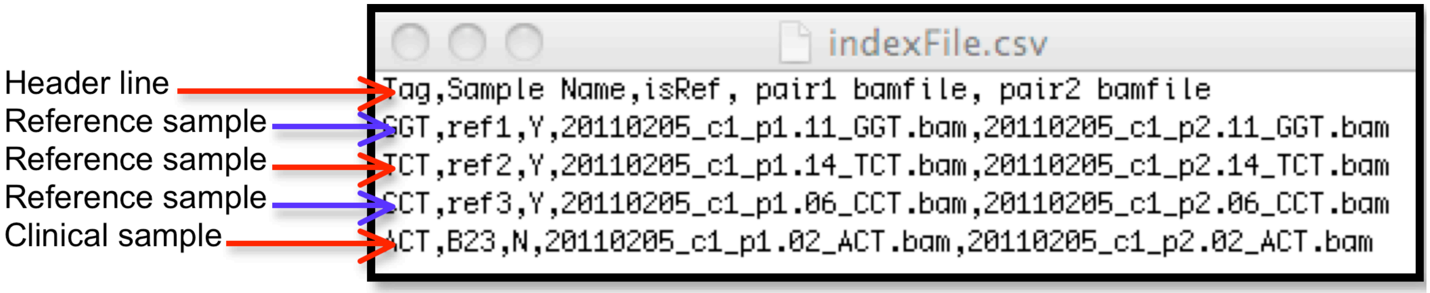


```
Tag,Sample Name,isRef, pair1 bamfile, pair2 bamfile
GGT,ref1,Y,20110205_c1_p1.11_GGT.bam,20110205_c1_p2.11_GGT.bam
TCT,ref2,Y,20110205_c1_p1.14_TCT.bam,20110205_c1_p2.14_TCT.bam
CCT,ref3,Y,20110205_c1_p1.06_CCT.bam,20110205_c1_p2.06_CCT.bam
ACT,B23,N,20110205_c1_p1.02_ACT.bam,20110205_c1_p2.02_ACT.bam
```

OUTPUT FORMAT

The algorithm output is a call table -- a comma-separated file with one line for each base position and each line in the following format:
AlginmentReferencePosition,AlignmentBase,Call,SecondBase,CenteredErrorPrc,ReferenceErrorPrc,SecondBasePrc

AlignmentReferencePosition: the relative position in the provided reference sequence
AlignmentBase: the reference base aligned to the given position
Call: 1 if a variant is called, 0 if no variant is called
SecondBase: the most frequent non-reference base
CenteredErrorPrc: the percent error of the sample relative to the percent reference error
ReferenceErrorPrc: the percent reference error calculated by the algorithm
SecondBasePrc: the percentage of reads that are the second base (not taking into account the inherent error at the position)

The call is made using only the hypothesis test. We recommend thresholding the CenteredErrorPrc to 0.05% or greater depending on the resolution desired to minimize false positive calls.

One output file is created for each sample name provided in your index file.
An example call table is shown below:

calltbl_1_B23.txt

AlignRefPos	AlignBase	Call	SecondBase	CenteredErrorPrc	ReferenceErrorPrc	SecondBasePrc
40	A	0	G	0.01967	0.05218	0.03864
41	A	1	G	0.10411	0.09011	0.16305
42	C	1	T	0.03421	0.05040	0.03856
43	C	0	T	0.00412	0.05851	0.03611
44	C	0	T	0.00748	0.05777	0.03179
45	T	1	C	0.07788	0.08992	0.13493

In addition to output call tables, a summary text file is created in the results directory that contains information about the estimated error rates in the sample set as calculated by the algorithm.
An example summary file is shown below:

summary_error.txt

```
Lane Summary Statistics
Total Variation = 4.0558e-08
Position-marginal variation (var): Var[E(r/n|pos)] = 3.7648e-08
Position-conditional variation (var): E[Var(r/n|pos)] = 2.9105e-09
H0: Equality between marginal and conditional p = 0.000e+00

Average error rate: 0.0007
Average standard deviation: 2.015e-04
Percentiles (5, 95): (0.0005, 0.0011)
Min and Max Error rates: (0.0003, 0.0019)
```

EXAMPLES
The web site for the software package provides sample data and configuration files as well as our output files for those runs so that you can validate your installation.

ADDITIONAL TOOLS
Get_Qual_Depth_Range_Slow.m
Calculating the base quality and depth ranges of the samples: A MATLAB program that is also available as a deployed MATLAB command line program, run_get_Qual_Depth_Range.sh, is provided that takes in as input a txt file with a list of Samtools pileup format files and outputs base quality minima, maxima, and averages as well as the pre-base quality filter and post-base quality filter minimum, maximum, and average depth for each pileup file in the dataset. It also computes an average of these values across all pileup files and thus across all samples included in the input data set.

Get_Qual_Depth_Range_Fast.m
Calculating the base quality and **approximate** depth ranges of samples. This program is identical to Get_Qual_Depth_Range_Slow but uses shortcuts to calculate depth that sacrifice the accuracy of depth

calculations in order to make the program significantly faster (x vs y). The base quality information calculated is accurate, but the total depth values produced from Get_Qual_Depth_Range_Fast may be off by as much as +/- 10 reads.

COMMON ERRORS

MCR required paths not properly installed

- Error will occur when deployed program is run from the command line when the program is looking for the MCR libraries.
- Follow instructions above for setting the correct environmental variables to prevent this problem

Wrong version of MCR or MATLAB referenced when running the program from the command line:

- Error will occur when deployed program is run from the command line when the program is looking for the MCR libraries
- Make sure you have MCR version 7.17 or MATLAB 2012a installed.
- Check the exact location of these files to make sure you have not inputted an incorrect path

Incorrect path to configuration/index files or data/results directories:

- Error will occur when trying to load sample information.
- Make sure to change the paths in the configuration file to reflect your current directory structure.
- The paths in the example configuration files provided reflect the directory structure on which they were originally run and will need customizing before they will work on your computer.
- If relative paths (../../data/example/BAM_files) are used instead of absolute paths (/mnt/tcga/rvd/data/example/BAM_files), they should be relative to the location from which RVD is called NOT the directory in which the program is stored or the directory of the configuration file.

Deployed program hangs up using nohup:

- MATLAB deployed programs are known to have some problems running through nohup
- Running these programs using

  echo ***command*** | at now

  is an alternative to nohup that works more consistently with MATLAB deployed programs.
