# Supplementary material for: RVD: a command-line program for ultrasensitive rare single nucleotide variant detection using targeted next-generation DNA resequencing
Source: BMC Res Notes. 2013 May 23;6:206. doi: 10.1186/1756-0500-6-206 (PMC3695852; doi:10.1186/1756-0500-6-206)
Supplement: Additional file 2 — Containing supplementary figures. [file 1756-0500-6-206-S2.pdf]

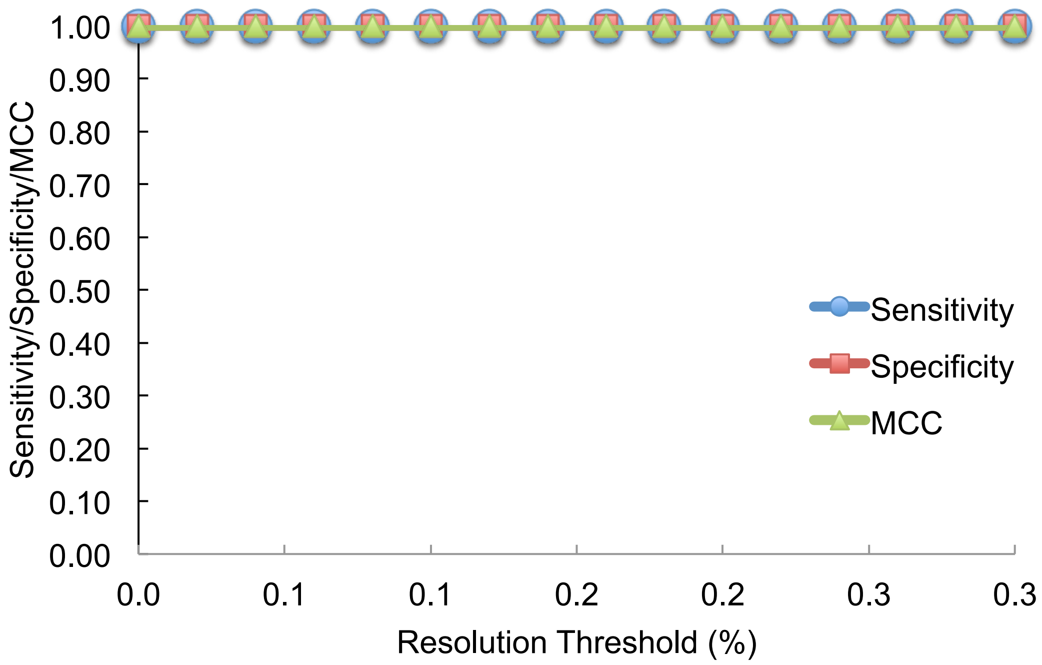

Figure S1 A. Resolution threshold scan for 0.3% variants.

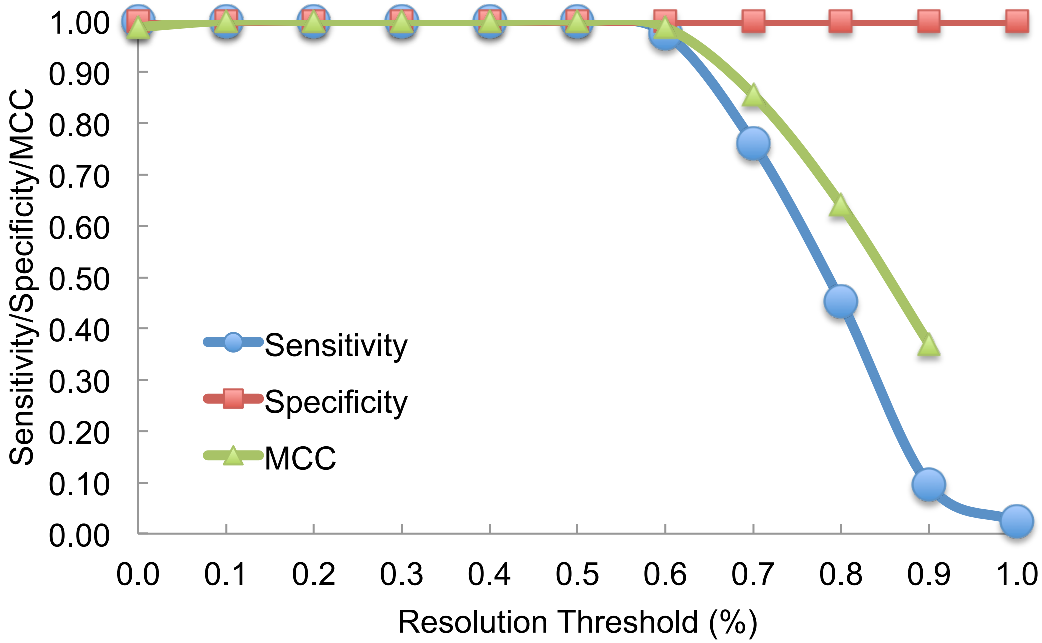

Figure S1 B. Resolution threshold scan for 1% variants.

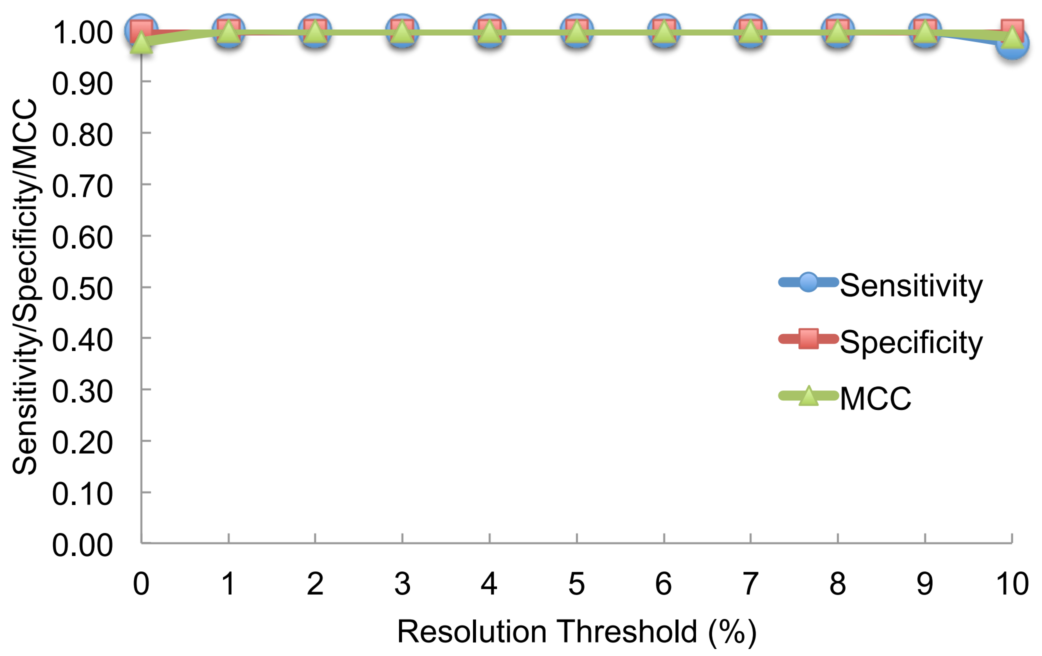

Figure S1 C. Resolution threshold scan for 10% variants.

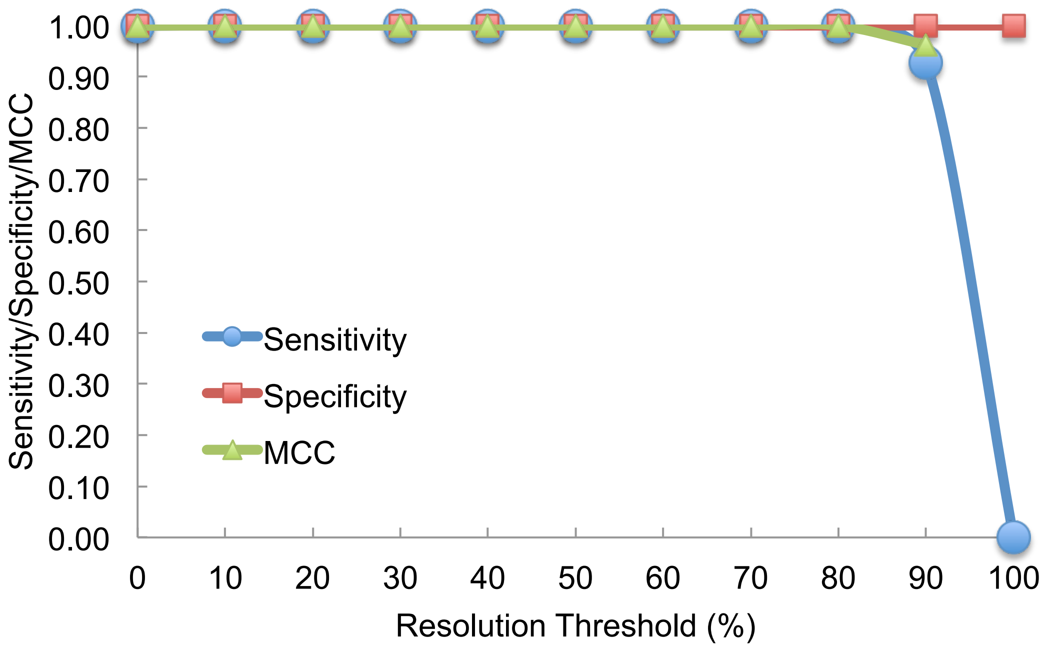

Figure S1 D. Resolution threshold scan for 100% variants.

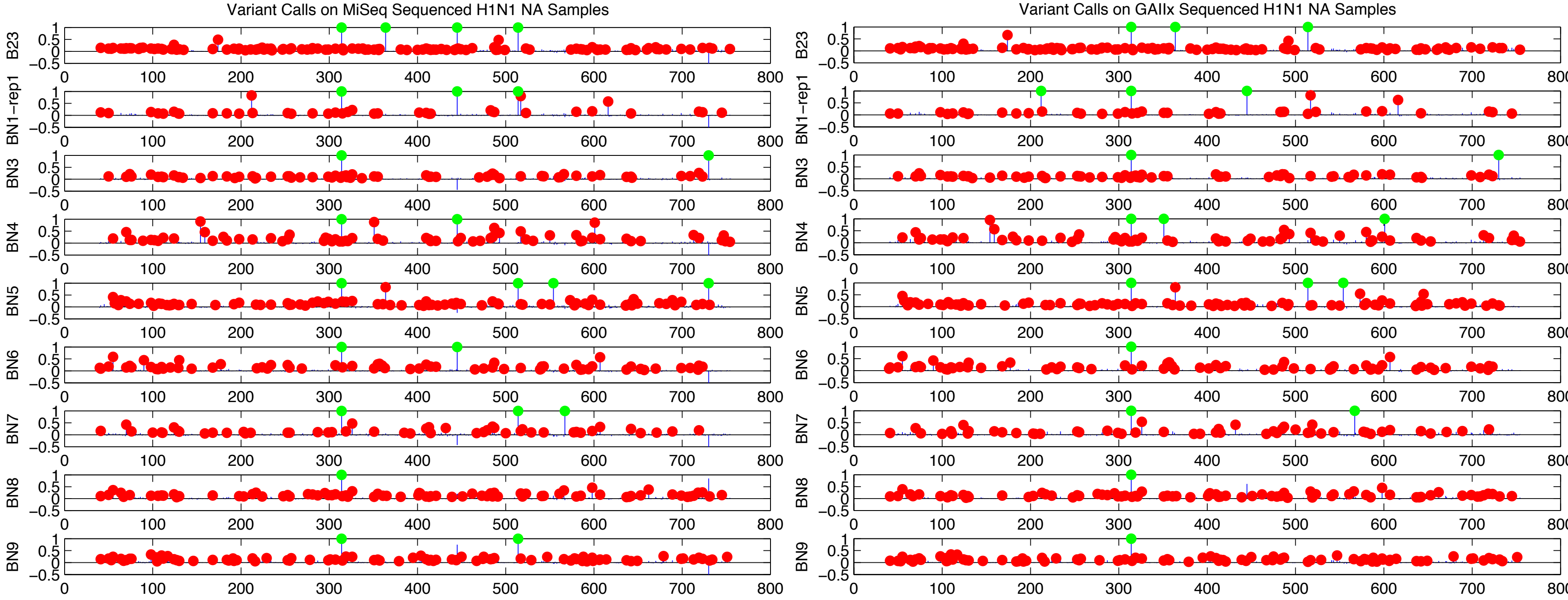

Figure S2. Variant calls on MiSeq and GAIIx H1N1 Neuraminidase Samples. The same sequencing library was run on the Illumina GAIIx and the MiSeq platforms with a base quality threshold of 30. While the MiSeq platform delivers 10x less sequencing data than the GAIIx, the application is still able to call variants at a minor allele fraction below 1% that were called on the GAIIx. Differences between the frequency of variants in the MiSeq and GAIIx are likely due to the significantly decreased depth on the MiSeq platform. This figure is an update on Figure 5 appearing in Flaherty et. al.'s publication in Nucleic Acid Research.

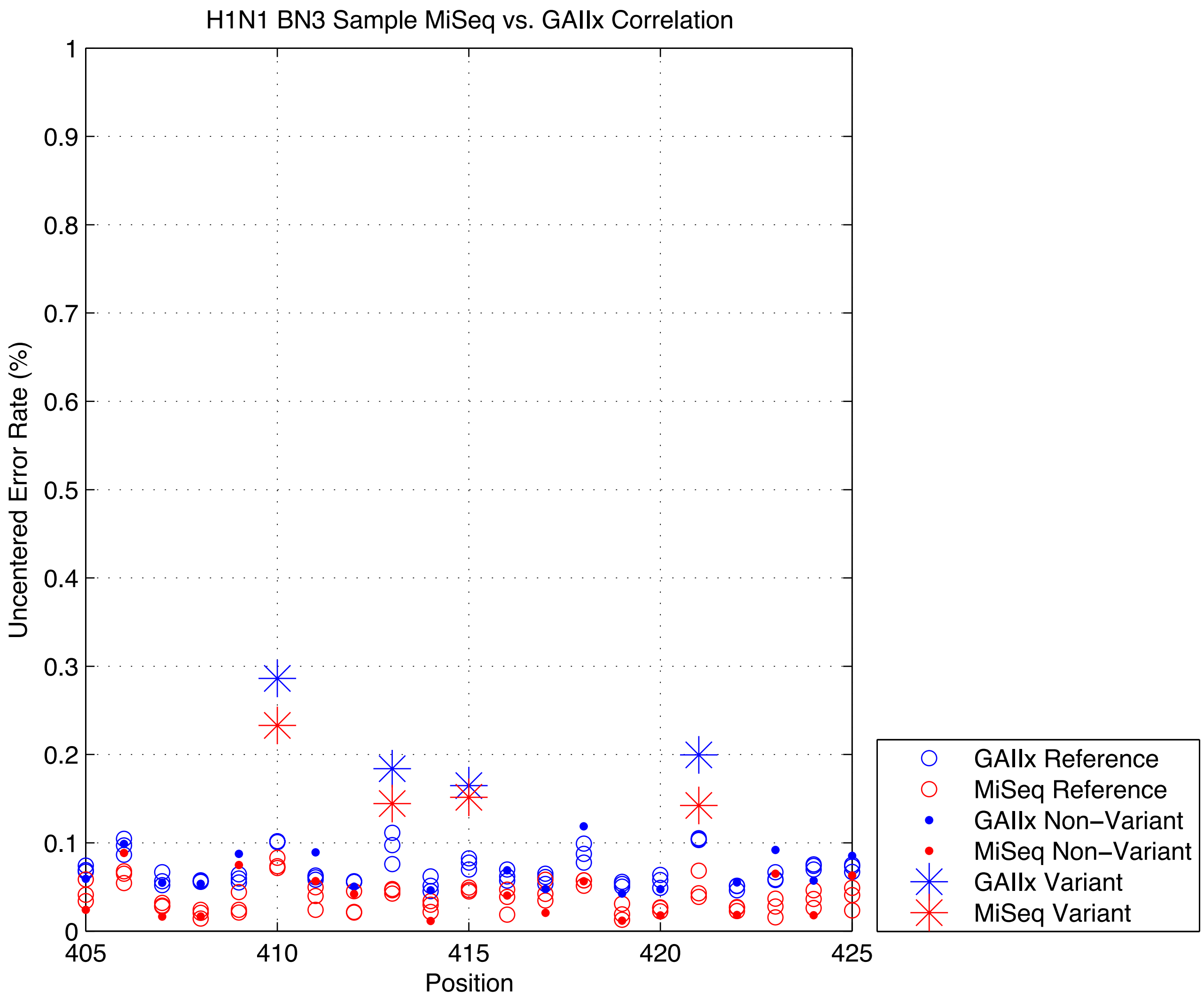

Figure S3. Correlation between calls on the MiSeq and GAIIx lane 1 calls. A detailed view of 21 bases in the neuraminidase gene in sample BN3 shows that there is correlation between the variant calls made on the MiSeq and the GAIIx. Four positions (410, 413, 415 and 421) were called on the both the MiSeq and GAIIx, and 17 positions were called as non-variant by both platforms.
